# Commentary: Does Severity of Alzheimer's Disease Contribute to Its Responsiveness to Modifying Gut Microbiota? A Double Blind Clinical Trial

**DOI:** 10.3389/fneur.2019.00667

**Published:** 2019-07-05

**Authors:** Friedrich Leblhuber, Kostja Steiner, Burkhard Schuetz, Dietmar Fuchs

**Affiliations:** ^1^Department of Gerontology, Neuromed Campus, Kepler University Clinic, Linz, Austria; ^2^Biovis Diagnostik MVZ GmbH, Limburg, Germany; ^3^Division of Biological Chemistry, Biocenter, Innsbruck Medical University, Innsbruck, Austria; ^4^Division of Medical Biochemistry, Biocenter, Innsbruck Medical University, Innsbruck, Austria

**Keywords:** Alzheimer's dementia (AD), probiotic supplementation, inflammation, TYM test, biomarkers

A. Agahi and colleagues in their study evaluated the responsiveness of inflammatory and oxidative biomarkers to a 12 weeks probiotic treatment in Alzheimer disease (AD) patients (1). After assessment of cognitive and serum biomarkers pre- and post-treatment in 96 patients with severe AD, they concluded that the cognitive Test Your Memory (TYM) test and markers like serum total antioxidant capacity, total glutathione, cytokines IL-6, IL-10, and TNF-α, and 8-hydroxy-2′-deoxyguanosine and plasma nitrite/nitrate were insensitive to probiotic supplementation. They concluded that severity of disease and time of administration seems to deeply affect results of treatment.

To evaluate the cognitive decline in their AD patients they applied the TYM test, which is described as suitable for detecting dementia earlier than the mini mental state examination (MMSE) (2). The TYM test is superior in discriminating between healthy controls and patients with mild cognitive impairment (MCI). To use the TYM test instead of the MMSE probably was the reason for the fact that they did not find any difference in cognition between the *verum* treated group and the placebo group of severely demented AD patients after 3 months of supplementation with probiotics. In Figure 2 as well as in Figure 3 of their study (1) they demonstrated a clearly significant difference between the TYM scores between controls and AD patients as well as between moderate AD and severe AD patients (both *p* < 0.0001). Regarding the possible influence of probiotic supplements, they probably had achieved more conclusive results (a) using the MMSE test instead of the TYM test and (b) comparing also normal individuals vs. patients with mild or moderate AD. Dysbiosis of intestinal microbiota in the elderly may cause leaky gut which results in silent systemic inflammation. Subsequent neuroinflammation may be a fundamental part of the pathomechanism in the early and preclinical course of AD (3).

In our own study (4) in a group of 20 AD patients with a more advanced stage of dementia (MMSE 18.5 ± 7.7), we did find changes of serum and fecal inflammatory parameters already after 4 weeks of supplementation of a multispecies probiotic (Omnibiotic Stress Repair®, Allergosan Graz Austria). The preparation consists of *Lactobacillus casei W56, Lactococcus lactis W19, Lactobacillus acidophilus W22, Bifidobacterium lactis W52, Lactobacillus paracasei W20, Lactobacillus plantarum W62, Bifidobacterium lactis W51, Bifidobacterium bifidum W23*, and *Lactobacillus salivarius W24* (7.5 ×10^9^ intestinal bacteria/per day). Inflammation-associated fecal zonulin was found significantly declining (*p* = 0.01) and anti-inflammatory *Faecalibacterium prausnitzii* significantly increased (*p* < 0.001) after 4 weeks of probiotic supplementation. By contrast, serum inflammation marker neopterin as well as kynurenine increased (*p* < 0.05), probably pointing to an activation of macrophages/dendritic cells by the probiotic intervention. The increase of nitrite levels from 238 ± 421 (mean±SEM) to 1050 ± 442 μmol/L was only slightly outside the level of significance (U = −1.423, *p* = 0.078; Mann-Whitney *U*-test). Notably, nitrite concentrations better than nitrite+nitrate levels mimic nitric oxide production (5). As a result, our recent study (4) shows—contrary to the series of Agahi et al.—that the supplementation of demented AD patients with a multispecies probiotic clearly influences gut bacteria composition. The correlation between neopterin concentrations and tryptophan breakdown as is reflected by the kynurenine to tryptophan ratio (Kyn/Trp) most probably was caused by the activation of macrophages due to probiotic supplementation for 4 weeks (4). We did not control the MMSE after such a short period of 4 weeks to avoid a measurement error due to the test-retest variability (6).

The question arises, why a 12 week's probiotic supplementation did not show effects neither on pro-inflammatory nor on anti-inflammatory markers in the patients studied by Agahi et al. (1). Was it the fact that they used only 3 ×10^9^ bacterial strains containing 3 bacteria compared to 7.5 ×10^9^ bacteria of 9 different strains supplemented daily in our study (4)?

The results of our exploratory study (4) can only be regarded as preliminary due to the small sample size and due to the lack of a placebo treated group. Larger populations including patients with MCI as well as cognitively normal subjects are necessary to show whether probiotics are able to influence cognitive function at the very beginning of pathogenic changes of neurodegeneration and AD dementia. Future intervention studies with prebiotics and probiotics should include measurement of lipopolysaccharide activity (7) and short chain fatty acids (8, 9) to elucidate their possible role in the pathogenesis of AD for better understanding the disease mechanisms. This would provide the evidence needed to create more effective therapeutic interventions in AD.

## Author Contributions

All authors listed have made a substantial, direct and intellectual contribution to the work, and approved it for publication.

### Conflict of Interest Statement

The authors declare that the research was conducted in the absence of any commercial or financial relationships that could be construed as a potential conflict of interest.
